# Spatiotemporal Effects of Supplementary Feeding of Wild Boar (*Sus scrofa*) on Artificial Ground Nest Depredation

**DOI:** 10.1371/journal.pone.0135254

**Published:** 2015-08-05

**Authors:** Ragne Oja, Karoline Zilmer, Harri Valdmann

**Affiliations:** Department of Zoology, Institute of Ecology and Earth Sciences, University of Tartu, Tartu, Estonia; University of Sassari, ITALY

## Abstract

Supplementary feeding of ungulates, being widely used in game management, may have unwanted consequences. Its role in agricultural damage is well-studied, but few studies have considered the potential for the practice to attract ground nest predators. Our goal was to identify the factors influencing ground nest predation in the vicinity of year-round supplementary feeding sites for wild boar and to characterise their spatiotemporal scope. We conducted two separate artificial ground nest experiments in five different hunting districts in south-eastern Estonia. The quantity of food provided and distance of a nest from the feeding site were the most important factors determining predation risk. Larger quantities of food resulted in higher predation risk, while predation risk responded in a non-linear fashion to distance from the feeding site. Although predation risk eventually decreases if supplementary feeding is ceased for at least four years, recently abandoned feeding sites still pose a high predation risk.

## Introduction

During recent decades, wild boar (*Sus scrofa*) abundance has increased throughout Europe [1–3]. The species can exploit various habitat types and their habitat use largely reflects food abundance [2,4–9]. In northern environments, where natural food is scarce, elevated wild boar population size is commonly associated with supplementary feeding [6,8,10]. In Estonia, supplementary feeding is widespread and increasing, with the number of supplementary feeding sites increasing twofold during the 2000s (Statistics Estonia; www.stat.ee). Supplementary feeding is carried out by hunters, who mostly offer grain, but also potatoes, apples, acorns and maize. However, providing large amounts of supplementary food can have undesirable effects. Some earlier studies have addressed the effect of supplemental food or prey on nest predation in various systems. In these studies, the effects of supplementary feeding ranged from decreased nest predation [11] to increased nest predation [12–14] or no effect [15–16]. To our knowledge, the effect of supplementary feeding of wild boar on ground nest depredation of free ranging birds has not previously been studied.

Nest predation is a significant factor in the population dynamics of forest grouse. In capercaillie (*Tetrao urogallus*), predation can account for 90% of chick mortality [17], and in the Italian Alps, which otherwise offers excellent habitat structure, reproductive success of capercaillie remains low due to predation [18]. Despite high levels of predation, black grouse (*Tetrao tetrix*) hens show high fidelity to nesting areas [19]. Along with red foxes (*Vulpes vulpes*) and several mustelids (*Mustelidae*), the wild boar is one of the most common mammalian predators of forest-dwelling grouse [20–22]. It has been shown to be responsible for the majority of nest losses in various artificial nest experiments in different habitats [23–25].

Thus, extensive supplementary feeding of wild boar can affect forest grouse in several ways. First, wild boars can be attracted to spend more time in grouse habitats by supplementary feeding [9,26]. Naturally, forest grouse prefer spruce-dominated habitats [27] and old bilberry forest [28], which in Estonia practically lack natural food for wild boar, but are preferred for day-time resting [9,29]. Although the majority of wild boar diet consists of plant material, animal food is consumed opportunistically [30–32], whereas the occurrence of chicks and nestlings is the highest in spring [30]. Second, supplementary feeding sites can attract other nest predators [12,13,24]. Wild boar feeding sites in Estonia provide a stable food source for the omnivorous invasive raccoon dogs (*Nyctereutes procyonoides*) [33]. Invasive species, unlike native predators, have the potential to increase depredation impact on forest-dwelling grouse [34,35]. Red foxes are also attracted to feeding sites, probably due to increased abundance of small mammals in proximity. Third, the high load of wild boar and other predators that are supported by extensive supplementary feeding can increase the overall predation impact on free-ranging birds [36].

Although the attraction of non-target species to feeding sites is well documented, there are few data about the spatiotemporal extent of the effect. The effect of increased nest predation in the vicinity of temporary feeding sites can exceed 225 m [14], but it does not reach 2 km [15]. Similarly, little is known about the lasting effects of supplementary feeding sites, when food is no longer provided and the impact of wild boar predation on free-ranging ground-nesting birds. The aim of our artificial nest experiment was to investigate the spatiotemporal factors that influence nest fate in the vicinity of active supplementary feeding sites and the continuing effects of abandoned sites. We predicted that:

- nest predation risk in the vicinity of active sites is determined by the quantity of supplementary food available, because large amounts of food may concentrate more potential nest predators;
- predation risk is negatively related to distance from the feeding site;
- nest depredation is similar in the vicinity of control plots and abandoned feeding sites, because wild boar should not be attracted to such sites when food is not present.

## Materials and Methods

### (a) Ethics Statement

No animals were harmed or captured in this study and the methods applied were non-invasive. The field study was conducted in public forest lands, managed by the State Forest Management Centre (www.rmk.ee). The field studies did not involve endangered or protected species. Although hazel grouse and black grouse were observed during fieldwork, the experiments took place in legal hunting districts, not protected areas. No additional supplementary food or baiting was provided for game animals by the authors, all supplementary feeding sites were maintained by local hunters.

### (b) Study areas

Our study area was located in south-eastern Estonia and included four hunting districts in Tartu County (Luunja, Kastre, Tähtvere, Võnnu) and one in Valga County (Valga). Year-round supplementary feeding of wild boar is carried out in all of these areas, although it is particularly intensive in winter. During the course of the experiment in late spring, feeding sites were divided into two categories–sites providing at least 50 kg of feed (large) and sites providing <25 kg (small) every week. The density of wild boar is supposed to be similar in these areas–hunters estimate the winter density to be 17–20 wild boar per 1000 ha of forested area (data from the Estonian Environment Agency, www.keskkonnaagentuur.ee). The Kastre and Valga study areas mainly consists of coniferous forest, while Luunja, Tähtvere and Võnnu are more varied with some mixed and deciduous forest as well. The dominant tree species in coniferous forest was Scots pine (*Pinus sylvestris*) with some Norway spruce (*Picea abies*) also present. Deciduous forest consisted of common aspen (*Populus tremula*) and birch *Betula* sp. and there were no mast trees. Mixed forest contained Norway spruce in addition to the common deciduous forest species. The forests in our study areas were of varying age classes – dominant trees were 20–140 years old.

### (c) Artificial nest placement

Two separate artificial nest experiments, each lasting three weeks, were conducted in the late spring of 2010, 2012 and 2013. Each nest consisted of three quail eggs placed into a small depression on the ground. The nests were put in place between 11 AM and 6 PM to limit disturbance to nocturnal mammals [37,38], which were considered to be the most frequent predators in the vicinity of supplementary feeding sites. Such timing may increase predation by birds [39], but we expected the influence of birds to be minor compared with that of mammals. No specific scent was added to attract predators, as absence of bird scent at a nest does not appear to hinder mammalian predators [40].

GPS coordinates of each nest site were recorded using Garmin GPSMAP 62 with a precision of ±3 m, and the nearest tree was marked with tape to facilitate relocation of nests. For each nest site we determined forest type: deciduous, coniferous or mixed forest or forest patch, where logging had recently occurred and the trees were 2–8 years old. Only eight nests in the first experiment were situated in such logged forest, but we decided to include this category, because forest grouse reproductive success can be high in logged areas [41]. Nests were checked once over a total period of three weeks, a nest was considered to be depredated if at least one of the eggs was missing or damaged [23,42].

### (d) Experimental design

**Fig 1:**
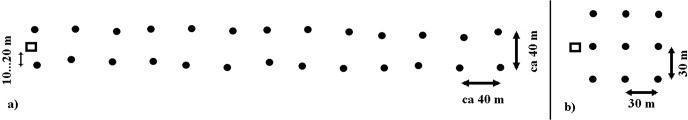
The first experimental design (a) and the adjusted design of the second experiment (b). Rectangles denote the location of the supplementary feeding site and dots correspond to nests in the vicinity of the site.

The first experiment was conducted to investigate the key factors associated with predation risk in the forest surrounding supplementary feeding sites. In May 2012, a total of 312 nests were placed in the vicinity of 12 supplementary feeding sites (see study plots and distances in S1 Table) in the Tähtvere and Valga study areas. Two transects were established at each study plot, starting in proximity to the feeding site and leading into the forest interior (Fig 1A). The first artificial nest was placed approximately 10–20 m from the feed, and each transect contained 13 nests with approximately 40 m between adjacent nests. Ground cover, defined as the proportion of herbaceous understorey plants excluding mosses, was determined for each nest site using the method suggested by Booth *et al*. [43]. We used an 8.0 MP Olympus SP-560UZ camera attached to a 1.5 m tripod to ensure that height from the ground was the same for all nest sites. The distance of a nest from the nearest feeding site and closest neighbour was determined with MapInfo Professional 10.5 using the GPS coordinates obtained during fieldwork.

The second experiment was conducted to investigate long-lasting effects of abandoned feeding sites. It started with a pilot study in 2010 and proceeded with additional experiments in 2012 and 2013. During the 3-year study period, a total of 306 nests were placed in the Luunja, Tähtvere, Kastre and Võnnu study areas in 31 different study plots (see locations in S2 Table). Three of the sites (one active and two abandoned) were observed during two years; others were observed during one year. For the pilot study, a total of nine nests were placed in each plot in a 3 by 3 pattern, with the central nest closest to the feeding site. In 2012 and 2013, the pattern remained the same, but due to the position of feeding sites with regard to the forest edge in the increased sample, nests were placed along one edge of the feeding site (Fig 1B), rather than placing them around the site. Supplementary feeding sites were categorised as active (12 sites) or abandoned (10 sites), if no feed had been supplemented for at least one year. Control plots (9 sites) were situated at similar distances from roads and forest edges [9,20] as supplementary feeding sites, but were located >500 m from the nearest active or abandoned feeding site.

### (e) Data analysis

In each experiment, nest depredation was modelled using generalized linear models with mixed effects (GLMM), where nest fate was a binary dependent variable (0 – nest survived; 1 – nest depredated). Study plot (SP, corresponding to a specific active or abandoned feeding site or control plot) was included as a random factor. Using study area or year of the experiment as an additional random factor did not improve the fit of the models (using corrected Akaike Information Criterion; AICc), and the confidence interval for the variance of the random effects contained zero. Therefore, the slight change in the design of the second experiment, as well as the problem of carrying out experiments in different years, is unlikely to have influenced the results. In order to determine which factors best predicted nest depredation risk in the vicinity of supplementary feeding sites, models with different independent variables were tested, and AICc [44] was used for model selection. To test whether variables should be retained, simplified models were compared to more complex models using the chi-square goodness-of-fit statistic.

In the first experiment, the dependent variables were: the quantity of available food (INT), distance from the feeding site (DIST), herbaceous plant cover (%; COV), forest type (FT), fate of the nearest neighbouring nest (0 – nest survived; 1 – nest depredated; NF) and study area (Tähtvere or Valga; SA). Interactions between DIST × COV, DIST × INT and COV × INT were also included. To investigate possible edge effects in the vicinity of supplementary feeding sites, the effect of distance from the feeding site was tested for non-linearity. We fit models containing linear, quadratic, cubic and quartic distance functions and used the AICc and chi-square statistics to test whether any of the more complicated non-linear models represented a significant improvement over the linear model. Additionally, in order to test, whether depredation events were independent, the relationship between nest depredation and the distance to the nearest neighbouring nest was tested. In the second experiment, the dependent variables were: type of the study plot (feeding site or control area; TYPE), period of non-use of the feeding site (time in years after the abandonment of a feeding site, “0” for both active feeding sites and control areas; AGE) and forest type (FT) as well as the interactions between AGE × FT and TYPE × FT. All statistical analyses were carried out with R 3.0.1 [45], using the package lme4 [46].

## Results

### (a) First experiment

In the first experiment, 224 out of 312 nests were depredated (72%). Nest depredation was independent of the proximity of the nearest neighbouring nest (p >0.05), suggesting depredation events were independent. The inclusion of interaction terms did not improve the linear model in terms of AICc (Table 1) or model fit (χ^2^_3_ = 1.68, p = 0.641). The model with the cubic function for DIST had a lower AICc score than the model containing the quartic function (Table 1) and was similar in terms of goodness-of-fit (χ^2^_1_ = 0.392, p = 0.531). However, the linear and quadratic function both resulted in significantly higher AICc scores and loss of goodness-of-fit (linear: χ^2^_2_ = 8.33, p = 0.016; quadratic: χ^2^_1_ = 8.22, p = 0.003). Hence, in all further simplified models without interactions, a cubic function of DIST was used. Nest predation did not depend on FT, NF or SA (p>0.05). Dropping these three variables in the first simplified model improved the model in terms of AICc (Table 1) and there was no loss of goodness-of-fit, when compared to the models with cubic function of DIST (χ^2^_4_ = 1.62, p = 0.804) or interaction terms (χ^2^_4_ = 1.58, p = 0.811).

**Table 1:** Selection of models predicting depredation in the vicinity of active wild boar feeding sites.

Model	K_i_	AICc_i_	∆_i_(AICc)	*ω*_i_(AICc)
**DPR = INT + DIST + DIST** ^**2**^ **+ DIST** ^**3**^ **| SP**	**6**	**296.02**	**0**	**0.4**
**DPR = INT + DIST + DIST** ^**2**^ **+ DIST** ^**3**^ **+ COV | SP**	**7**	**296.89**	**0.87**	**0.3**
**DPR = INT| SP**	**3**	**297.72**	**1.70**	**0.2**
DPR = INT + COV | SP	4	298.51	2.49	0.1
DPR = INT + DIST + COV | SP	5	300.52	4.50	<0.1
DPR = INT + DIST + DIST^2^ + DIST^3^ + COV + FT + NF + SA | SP	11	303.78	7.76	0
DPR = INT + DIST + COV + DIST × INT + COV × INT + DIST × COV | SP	8	305.12	9.10	0
DPR = INT + DIST + DIST^2^ + DIST^3^ + DIST^4 +^ COV + FT + NF + SA | SP	12	305.54	9.53	0
DPR = INT + DIST + COV + FT + NF + SA | SP	9	307.84	11.82	0
DPR = INT + DIST + DIST^2^ + COV + FT + NF + SA | SP	10	310.45	14.44	0
DPR = INT + DIST + COV + FT + NF + SA + DIST × INT + COV × INT + DIST × COV | SP	12	312.10	16.09	0
DPR = DIST + DIST^2^ + DIST^3^ | SP	5	346.84	50.82	0
DPR = DIST + DIST^2^ + DIST^3^ + COV | SP	6	348.22	52.20	0

The best models are in bold. K_i_−number of estimated parameters for model i; *∆*_i_(AICc) = [AICc_i_−min(AICc)]; *ωi*(AICc)–AICc weights. DPR–nest fate, binomial variable (1 – nest depredated, 0 – nest survived); INT–feeding site category, large (>50 kg feed present) or small (<25 kg feed present); DIST–distance from the feeding site; COV–ground cover; FT–forest type (mixed, coniferous or logged forest); SA–study area (Tähtvere or Valga), NF–fate of the nearest neighbouring nest (1 – nest depredated, 0 – nest survived); | SP–study plot locality (specific supplementary feeding site), where | denotes the random effect; × stands for interaction

Based on AICc values and weights, three good models could be distinguished (Table 1): a) INT + DIST + DIST^2^ + DIST^3^ + COV, b) INT + DIST + DIST^2^ + DIST^3^ and c) INT. The factor INT was included in each of the best models and the removal of INT resulted in models with the lowest AICc scores. Model b) had a slightly lower AICc than model a), but both were similar in terms of goodness-of-fit (χ^2^_1_ = 1.21, p = 0.271). Therefore, COV was dropped and the simpler model preferred. Model c) had a slightly higher AICc score and it differed significantly in terms of goodness of fit from model b) (χ^2^_3_ = 7.86, p = 0.049). The final simplification of dropping distance from the feeding site from the model was not accepted, and model b) was preferred to model c).

**Fig 2:**
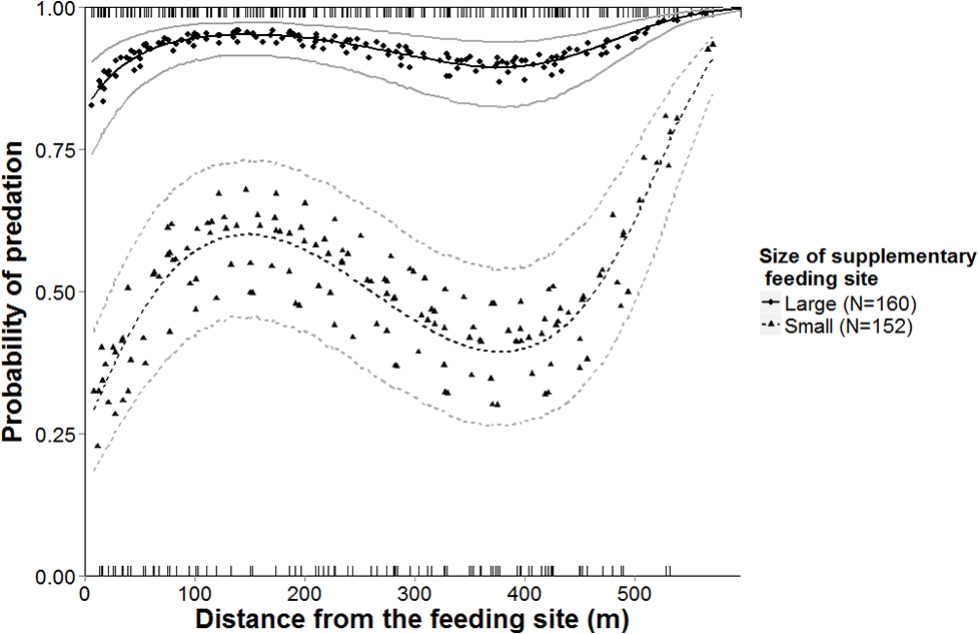
Predicted probability of nest depredation in relation to the amount of food and distance from the feeding sites. Predictions (EBLUPs, denoted as dots or triangles) and estimation (denoted as lines) of predation risk for artificial ground nests, according to the best GLMM in the first experiment. Nest fate is measured on a binomial scale (0 – nest survived, 1 – nest depredated); notches at the top and bottom of the figure indicate the distance of nests from wild boar feeding sites in the experiment and their fate. The solid black line indicates estimated predation risk for nests in the vicinity of large feeding sites (>50 kg of feed present); the dashed black line represents estimated predation risk for nests in the vicinity of small feeding sites (<25 kg of feed present), grey lines denote ± 1 SE.

According to the best model, nest depredation was significantly less frequent in the vicinity of small feeding sites compared to large sites (50.0% and 92.5% of depredated nests respectively; β = -2.563, SE = 0.394, p < 0.001). Models with distance from the feeding site as an explanatory variable were good only if INT was also included (Table 1). Nest depredation increased up to approximately 150 m (local maximum) and decreased thereafter, being lowest at approximately 380 m (local minimum) from the feeding site (Fig 2). Further distance from the feeding site led to an abrupt increase in depredation–nearly all of the nests were depredated.

### (b) Second experiment

**Fig 3:**
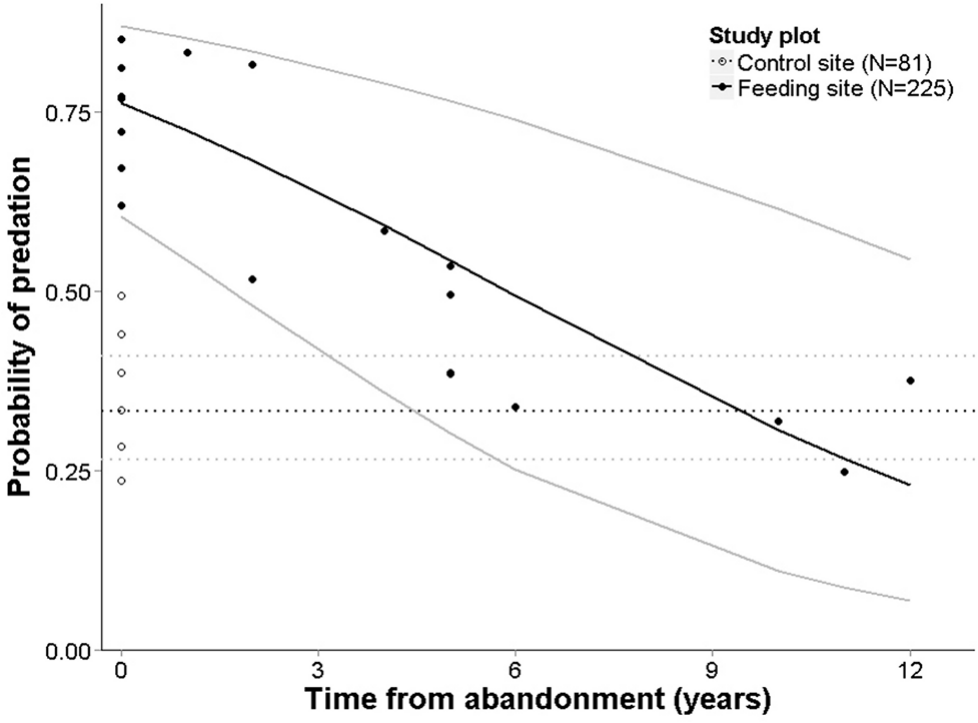
Depredation risk depends upon the period of non-use of an abandoned supplementary feeding site. Depredation risk in the vicinity of active or abandoned supplementary feeding sites – results of the best GLMM. Predicted depredation risk at control sites is marked with a dotted line (AGE = 0 in the model), grey lines denote ± 1 SE.

A total of 168 out of 306 artificial nests were depredated (55%), 75% and 48% in the vicinity of active and abandoned feeding sites respectively and 38% in control plots. TYPE and AGE produced the best model according to AICc (Table 2). According to the best model, depredation risk was significantly higher in feeding sites than in control plots (β = 1.854, SE = 0.415, p < 0.001) and decreased at abandoned feeding sites with increasing period of non-use (β = -0.197, SE = 0.054, p < 0.001), but was significantly higher during the first few years after abandonment (Fig 3). Model fit was not improved by the inclusion of forest type as an additional additive variable (χ^2^_2_ = 0.08, p = 0.962) or interaction terms in the model (χ^2^_6_ = 3.33, p = 0.767).

**Table 2:** Selection of models predicting depredation in the vicinity of active and abandoned wild boar feeding sites.

Model	K_i_	AICc_i_	∆_i_(AICc)	*ω*_i_(AICc)
**DPR = TYPE + AGE | SP**	**4**	**380.51**	**0.00**	**0.8**
DPR = TYPE + AGE + FT | SP	6	384.58	4.07	0.1
DPR = TYPE + AGE +FT + AGE × FT | SP	8	386.62	6.11	< 0.1
DPR = TYPE + AGE +FT + TYPE × FT | SP	8	387.89	7.27	< 0.1
DPR = TYPE + AGE +FT + AGE × FT + TYPE × FT| SP	10	389.79	9.27	0
DPR = TYPE + FT | SP	5	393.52	13.01	0
DPR = AGE + FT | SP	5	397.96	17.45	0

AICc was used for model selection; the best model is in bold. Ki−number of estimated parameters for model i; ∆i(AICc) = [AICci−min(AICc)]; ωi(AICc)–AICc weights. DPR–nest fate, binomial variable (1 – nest depredated, 0 – nest survived); TYPE – type of the study plot (supplementary feeding site or control plot), AGE – time in years that has passed from abandonment of a supplementary feeding site, AGE = 0 for active feeding sites and control plots; FT – forest type (deciduous, mixed or coniferous forest); | SP – study plot locality (supplementary feeding site or control area), where | denotes the random effect; × stands for interaction

## Discussion

The quantity of supplemental food provided at wild boar feeding sites was the most important factor predicting nest predation in our experiment. This effect occurred at a scale of up to 600 m from the feeding site, and not just in the immediate vicinity of the site. The effect of supplementary feeding appeared to diminish the effects of other factors, including nest cover, which has been emphasized elsewhere [11,12,22]. Similarly, supplemental food can minimize the impact of other factors—primarily nest density—on nest success [13]. In a study, where no consistent effect of nearest-neighbour fate or density on the success of artificial or natural ground nests was found [47], it was pointed out that this is to be expected when alternative prey (such as the supplemental food in our study) is abundant and predation occurs only accidentally. The effects of other factors can exceed the effect of supplementary feeding, when they reach extreme levels (as in [12]), but this was not the case in our study.

Nest depredation did not decrease linearly with distance. Compared to the forest interior, supplementary feeding sites contain a large amount of easily accessible food, which attracts wild boar and various non-target species, including small mammals, foxes and raccoon dogs. At the same time, these sites are commonly located in open areas, which pose a threat to visiting mammals and are generally avoided by wild boar [1]. Wild boar check the surroundings for threats prior to entering feeding sites [48], and the increase in nest depredation we observed close to feeding sites is probably the result of increased rooting activity while boar peruse for potential danger. Depredation increased again abruptly beyond 380 m from the feeding site, which may result from the overall high density of possible nest predators (72% of nests destroyed in the first experiment) or be caused by wild boar selecting bedding sites at an optimal distance from the feeding site. Wild boar occur more frequently in forest than in open areas and typically alter activity patterns on a small scale [49]. If wild boar are undisturbed and easily accessible supplemental food is present, bedding sites can be found less than 0.5 km from the feeding sites [50,51].

The relatively weak nature of the relationship between ground cover and nest depredation could potentially limit the effectiveness of anti-predator behaviour in forest dwelling grouse. Hazel grouse nest survival rates increase with understory cover [52], but by selecting visually concealed sites, grouse become exposed to olfactory predators [53]. A similar effect has been found in black grouse, who become more vulnerable to predation by goshawks when they attempt to avoid terrestrial predators [54]. Hence, it is possible that by choosing sites with good microhabitat parameters for the avoidance of mammalian predators in the vicinity of supplementary feeding sites, forest dwelling grouse become more vulnerable to bird depredation.

Our study shows that abandonment of a supplementary feeding site might not result in an immediate increase in forest grouse breeding success. The depredation rate remained high in the vicinity of recently abandoned sites; this could be due to older animals remembering a previous food source. Trials with domestic pigs demonstrate the use of memory for locating food [55, 56]. The mean age of wild boar in Estonia is about 2 years, which is consistent with the behaviour of older animal driving the lag in predation rates. If no longer sustained by supplemental food, visiting animals may even increase their searching activity in the vicinity of the abandoned feeding site, leading to short-term increase in depredation.

Our results accentuate the risks that the widespread practice of supplementary feeding poses on both management and conservation. We documented a significant increase of predation risk in the vicinity of active and recently abandoned supplementary feeding sites, whereas the effect depends positively on the amount of food available rather than ground cover or forest type. Both wild boar and other predators are attracted to feeding sites and surrounding forest. Moreover, the effect of increased predation risk does not disappear immediately after a feeding site has been abandoned. Therefore, we would recommend stopping supplementary feeding in sensitive areas and choosing feeding sites more carefully.

## Supporting Information

S1 Table Dataset of the first experiment. (XLSX)

S2 Table Dataset of the second experiment. (XLSX)
